# Cirrhosis Hampers Early and Rapid Normalization of Natural Killer Cell Phenotype and Function in Hepatitis C Patients Undergoing Interferon-Free Therapy

**DOI:** 10.3389/fimmu.2020.00129

**Published:** 2020-02-25

**Authors:** Elena Perpiñán, Sofía Pérez-Del-Pulgar, María-Carlota Londoño, Zoe Mariño, Concepción Bartres, Patricia González, Mireia García-López, Elisa Pose, Sabela Lens, Mala K. Maini, Xavier Forns, George Koutsoudakis

**Affiliations:** ^1^Liver Unit, Hospital Clinic, University of Barcelona, IDIBAPS, Barcelona, Spain; ^2^Centro de Investigación Biomédica en Red de Enfermedades Hepáticas y Digestivas, Barcelona, Spain; ^3^Division of Infection and Immunity, University College London, London, United Kingdom

**Keywords:** hepatitis C virus, natural killer cells, direct-acting antivirals, immune restoration, cirrhosis

## Abstract

**Background:** Chronic hepatitis C virus (HCV) infection impairs natural killer (NK) cell phenotype and function. Whether restoration of NK cells occurs after successful interferon (IFN)-free therapies remains a controversial issue.

**Aim:** To analyze how HCV-related liver cirrhosis impacts changes in NK cells prior and post-IFN-free therapies.

**Methods:** NK cell analysis by multicolor flow cytometry was performed in HCV-infected patients with (*n* = 17) and without (*n* = 14) cirrhosis at baseline, week 4 during therapy, and weeks 12 and 48 after the end of therapy (FU12 and FU48, respectively). Non-HCV cirrhotic patients (*n* = 12) and healthy individuals (*n* = 12) served as controls.

**Results:** At baseline, HCV cirrhotic patients presented an altered distribution of NK subsets (CD56^dim^ and CD56^bright^) with higher expression of NKp46, HLA-DR, NKp30, KIR2DL2/L3, NKG2A, and CD85j receptors compared to healthy controls. All frequencies normalized by FU48, except for CD85j^+^ cells. Likewise, substantial alterations were detected in NK cell function assessed by (i) signal transducer and activator of transcription 1 (STAT1) and phosphorylated levels of STAT1 and STAT4, (ii) degranulation (CD107a), (iii) cytotoxicity [tumor necrosis factor-related apoptosis-inducing ligand (TRAIL)], and (iv) cytokine production [IFN-γ and tumor necrosis factor-α (TNF-α)]. Of note, NK cell function at FU48 remained partially impaired. In contrast, non-cirrhotics showed normal baseline frequencies of HLA-DR-, NKG2A-, and CD85j-expressing NK cells. Importantly, altered baseline frequencies of NK cell subsets and NKp46^+^ CD56^dim^ cells, as well as NK cell function, were rapidly and completely restored.

**Conclusions:** NK cell phenotype alterations persist after HCV eradication in cirrhotic patients, while their function is only partially restored, compromising immune restoration and immunosurveillance.

## Introduction

Chronic hepatitis C virus (HCV) infection is associated with persistent liver inflammation, which progresses to cirrhosis in ~10–20% of patients over a period of 20–30 years (1). Cirrhosis compromises the capacity of the liver to maintain systemic immune homeostasis (2). Additionally, constant antigen exposure, among other mechanisms, can cause immune exhaustion (3). The advent of potent interferon (IFN)-free regimens against HCV infection (4, 5) provides the perfect setting to delineate whether rapid and complete viral eradication can drive immune reconstitution. The latter is particularly relevant in the setting of cirrhosis-related immune dysfunction.

Natural killer (NK) cells, which are key elements of the innate immune system, acquire an altered phenotype and function during chronic HCV infection (6, 7). These alterations include disturbed distribution of NK cell subpopulations (CD56^dim^ and CD56^bright^, the two main subsets according to the expression of the neural cell adhesion molecule CD56), distinct receptor expression, and polarization of NK cell function away from cytokine production toward cytotoxicity (8–10). In the wake of successful IFN-free therapies, three studies reported rapid on-treatment restoration (by weeks 8 and 12) of NK cell phenotype and function. This consisted of normalization of (i) NK cell receptor expression (NKp30, NKp46, HLA-DR, CD85j, and NKG2A), (ii) effector function [expression of tumor necrosis factor-related apoptosis-inducing ligand (TRAIL)], and (iii) IFN-α-induced response (expression of the degranulation indicator CD107a and phosphorylation of signal transducer and activator of transcription 1-alpha/beta, pSTAT1) (11–13). Of note, these studies included patients with mild to severe fibrosis without analyzing the impact of liver disease severity on NK cell normalization. Nakamura et al. (14) recently reported similar functional restoration between two groups of HCV patients with different fibrosis-4 index score. Nevertheless, the majority of patients had mild fibrosis, and the result was based on a single creatinine release assay, which cannot reflect the complex profile of NK cells. Other recent data have demonstrated that NK cell cytotoxicity, IFN-γ production (15), and the NK cell repertoire (16) remained altered after virus clearance albeit without patient stratification according to fibrosis scores.

Herein, we evaluate changes in NK cell phenotype and function induced by viral eradication in a well-defined patient cohort, with or without established liver cirrhosis, during and after successful direct-acting antiviral (DAA) therapies. We show that there exist substantial baseline alterations in NK cell phenotype and function in HCV patients with cirrhosis in comparison with healthy individuals, which are not fully reflected in cirrhotic patients of other etiologies. Furthermore, unlike in non-cirrhotic HCV patients, NK cell phenotype normalization does not occur immediately after viral eradication in patients with cirrhosis. Comparing with the profile of healthy controls, we find that TRAIL expression, along with *in vitro* phosphorylation of STAT4 and cytokine production, is not completely recovered in HCV cirrhotic patients, even 1 year after end of therapy. In contrast, HCV non-cirrhotic patients have less marked NK cell alterations at baseline, the majority of which are restored either during or at an early stage post-therapy. These data suggest that HCV infection induces a sustained imprint on NK cells in patients who have progressed to cirrhosis, compromising immune restoration despite viral clearance.

## Materials and Methods

### Study Cohort

Thirty-one patients with chronic HCV infection and treated with DAA regimens at the Hospital Clinic in Barcelona were prospectively included in the study. The first group consisted of 17 HCV cirrhotic patients (Model of End-Stage Liver Disease score <16), and the second group included 14 HCV non-cirrhotic patients. Peripheral blood samples were collected longitudinally before (baseline, B), at week 4 during therapy (W4), and at weeks 12 and 48 after the end of therapy (FU12 and FU48, respectively). HCV patients were predominantly men as described (17). All patients achieved a sustained virologic response (SVR) defined as undetectable HCV RNA 12 weeks after treatment completion. Exclusion criteria were the presence of hepatitis B virus or human immunodeficiency virus coinfection and previous liver transplantation. Viral load was determined as part of the clinical diagnostic. Samples obtained from 12 non-HCV cirrhotic patients [seven with alcoholic cirrhosis and five non-alcoholic steatohepatitis (NASH)-related cirrhosis] and 12 healthy individuals, matched by age and sex, served as two distinct control sets (Table 1). Written informed consent was obtained from all patients, and the study was conducted according to the local regulatory requirements and ethics committee, Good Clinical Practice guidelines, and the Declaration of Helsinki.

**Table 1:** Baseline characteristics of the study cohorts.

**Parameters**	**Cirrhotic HCV** **(*n* = 17)**	**Non-cirrhotic HCV** **(*n* = 14)**	**Cirrhotic non-HCV** **(*n* = 12)**	**Healthy** **(*n* = 12)**	***P***
Age	66 (47-83)	57 (32-78)	63 (49-70)	61.5 (40-72)	n.s.
Male	11 (64.7%)	6 (42.8%)	8 (66.7%)	8 (66.7%)	n.s.
Genotype 1	15 (88.2%)	12 (85.7%)	n.a.	n.a.	n.s.
HCV RNA (Log IU/ml)	6.0 (4.2–7.4)	6.4 (5.9–6.9)	n.a.	n.a.	n.s.
FibroScan (kPa)	15.8 (9.3–35.8)	5.4 (3.1–10.3)	31.8 (13.8–56)	n.a.	<0.0001
Child-Pugh score	5 (5-7)	n.a.	6 (5-9)	n.a.	n.s.
MELD	8 (6-14)	n.a.	9 (7-13)	n.a.	n.s.
ALT (U/L)	88 (43-315)	49 (37-113)	38 (20-86)	n.a.	0.0005
AST (U/L)	73 (34-225)	47.5 (26-90)	34 (12-102)	n.a.	0.0003
GGT (U/L)	92 (24-217)	58 (9-373)	89 (23-310)	n.a.	n.s.
Platelet count (10^9^/L)	144 (83–272)	194 (142–296)	128.5 (63–201)	n.a.	0.0008
WBC count (10^9^/L)	6.3 (4.1–14)	5.6 (4.3–8.3)	6.1 (3.2–10.3)	n.a.	n.s.
Previous IFN-based therapy	9 (52.9%)	5 (35.7%)	n.a.	n.a.	n.s.
Treatment duration			n.a.	n.a.	n.s.
12/24 weeks	13 (76%)/4 (24%)	14 (100%)/0			
DAA treatment			n.a.	n.a.	0.004
SOF + LDV + RBV	5 (29.4%)	0			
SOF + LDV	0	4 (28.6%)			
SOF + DCV + RBV	2 (11.8%)	0			
SOF + VEL	0	1 (7.1%)			
PTV/r/OBV + DSV + RBV	5 (29.4%)	0			
PTV/r/OBV + DSV	5 (29.4%)	7 (50%)			
EBR/GZR	0	2 (14.3%)			

*n.a., not applicable; n.s., not significant; ALT, alanine aminotransferase; AST, aspartate aminotransferase; GGT, gamma-glutamyl transferase; DAA, direct-acting antiviral; IFN, interferon; MELD, Model for End-Stage Liver Disease; SOF, sofosbuvir; RBV, ribavirin; LDV, ledipasvir; DCV, daclatasvir; VEL, velpatasvir, PTV/r/OBV, paritaprevir/ritonavir/ombitasvir; EBR, elbasvir; GZR, grazoprevir; VOX, voxilaprevir; WBC, white blood cells; DSV, dasabuvir*.

Liver fibrosis was assessed by transient elastography [FibroScan (FS)]. Cirrhosis was diagnosed by a FS value >14 kPa (18) (*n* = 3/20, 15%), evident ultrasonographic signs of cirrhosis (19) (*n* = 6/20, 30%), or both (*n* = 11/20, 55%).

### Isolation and Storage of Peripheral Blood Mononuclear Cell and Serum Samples

Peripheral blood mononuclear cells (PBMCs) were separated from EDTA-anticoagulated blood on Ficoll Histopaque density gradients as previously described (20) and cryopreserved. For sera isolation, the patients' blood was collected in Vacutainer® Rapid Serum Tube (Becton Dickinson, Franklin Lakes, NJ, USA), separated after centrifugation at 2,500 × g for 10 min and kept at −80°C.

### Serological Analyses

Serum HCV-RNA was measured using the Siemens Versant Quantitative assay (Siemens Healthineers, Erlangen, Germany) with a lower limit of detection of 15 IU/ml. Cytokine IFN-α was quantitated in serum using the human pan IFNα enzyme-linked immunosorbent assay (STEMCELL Technologies, Vancouver, Canada), according to manufacturer's instructions.

### Natural Killer Cell Analysis

Cryopreserved PBMCs were thawed in Roswell Park Memorial Institute (RPMI)-1640 + GlutaMAX™ medium supplemented with 10% fetal bovine serum (FBS), 1% penicillin/streptomycin, and 1% sodium pyruvate (RPMI complete, all from Thermo Fisher Scientific, Waltham, MA, USA). Thawed PBMCs were stained either immediately or after an incubation period according to the protocols described below, prior to analysis on a BD FACSCanto™ II flow cytometer using FACSDIVA™ Software V 8.0.1 (BD Biosciences, San Jose, CA, USA) and FlowJo V10 (Tree Star Inc., Ashland, OR, USA). A complete list of antibodies used in this study is provided in Supplementary Table 1. Gates for positivity in multicolor panels were determined by fluorescence-minus-one control stains, as recommended (21).

#### Frequency and Expression of Activating and Inhibitory Receptors in CD56^dim^ and CD56^bright^ Natural Killer Subsets

PBMCs were stained for 30 min at 4°C in PBS + 2% FBS with LIVE/DEAD™ Fixable Aqua Dead Cell Stain Kit (Thermo Fisher Scientific, Waltham, MA, USA); anti-human lineage cocktail 3 (CD3, CD14, CD19, and CD20); and anti-CD56-PE, anti-NKp30-AlexaFluor647, anti-NKp46-PeCy7, anti-KIR2DL2/3-PerCPCy5.5 or anti-CD56-PerCPCy5.5, anti-HLA-DR-APC/Cy7, anti-CD85j-AlexaFluor647, or anti-NKG2A-PE. Thereafter, cells were fixed with PBS + 4% paraformaldehyde (PFA, Sigma-Aldrich, St. Louis, MI, USA).

#### Natural Killer Cell Degranulation Directly *ex vivo*

PBMCs were thawed and cultured overnight in RPMI complete medium at a concentration of 1–3 × 10^6^ PBMCs/ml. The following day, 0.5 × 10^6^ viable PBMCs were cultured during 6 h in the presence of anti-CD107a-PacificBlue with or without K562 (PBMC:K562 ratio 1:1) (ATCC, Manassas, VA) as described (9) without addition of cytokines and then stained with the LIVE/DEAD™ Fixable Aqua Dead Cell Stain Kit, anti-human lineage cocktail 3, and anti-CD56-PE as described above. Finally cells were fixed with PBS + 4% PFA.

#### STAT1, pSTAT1, and pSTAT4 Staining

PBMCs were thawed and cultured overnight as described above. The following day, 1.0 × 10^6^ viable PBMCs were stimulated with or without 300 ng/ml of IFN-α2 (BioLegend, San Diego, CA, USA) in RPMI complete medium for 20 min at 37°C and agitation at 300 rpm. During the stimulation period, cells were stained with LIVE/DEAD™ Fixable Aqua Dead Cell Stain Kit, anti-human lineage cocktail 3, and anti-CD56-V450. Thereafter, cells were fixed with the BD Cytofix Fixation Buffer for 30 min at 4°C. Then, cells were washed twice and resuspended in PBS supplemented with 2% FBS. Finally, cells were stained with anti-STAT1-PE and anti-pSTAT1 (pY701)-PerCPCy5.5 or anti-pSTAT4(pY693) during 60 min at room temperature.

#### Cytokine Production

PBMCs were thawed and cultured overnight as described above prior to stimulation with either IL-12 (0.5 ng/ml) or IL-15 (20 ng/ml, both purchased from BioLegend, San Diego, CA, USA) with or without K562 cells (PBMC:K562 ratio 2:1) as described (11). Cells were then washed and stained with anti-human lineage cocktail 3 and anti-CD56-PE as explained above. Cells were washed again, fixed, and permeabilized with the Cytofix/Cytoperm Kit (BD Biosciences, San Jose, CA, USA) and stained with anti-IFN-γ-PerCPCy5.5 and anti-TNFα-AlexaFluor647.

#### Tumor Necrosis Factor-Related Apoptosis-Inducing Ligand Staining Directly *ex vivo*

PBMCs were thawed and stained for 30 min at 4°C in PBS + 2% FBS with anti-human lineage cocktail 3, anti-CD56-PE, and anti-TRAIL-APC. Thereafter, cells were fixed with PBS + 4% PFA.

### Statistics

Statistical analyses were performed in GraphPad Prism (La Jolla, CA, USA) and SAS 9.4 software (Cary, NC, USA) as indicated in each figure legend. Statistical tests used were the following: Fisher's exact test for testing differences between groups in categorical variables; Mann–Whitney non-parametric test for comparisons of continuous variables between groups at different time points (B, W4, FU12, and FU48); and mixed models for repeated measurements (MMRM) for longitudinal analysis, including in the model the group effect, time, and the group by time interaction terms, declaring time as a categorical variable. The level of significance was established at the two-sided 5% level. Normalization was assessed by Mann–Whitney test comparing continuous variables between healthy individuals and HCV patients (cirrhotic or non-cirrhotic) at the different time points.

## Results

### Natural Killer Cell Phenotype Alterations Persist in Hepatitis C Virus Patients With Cirrhosis Several Months After Viral Eradication

**Figure 1:**
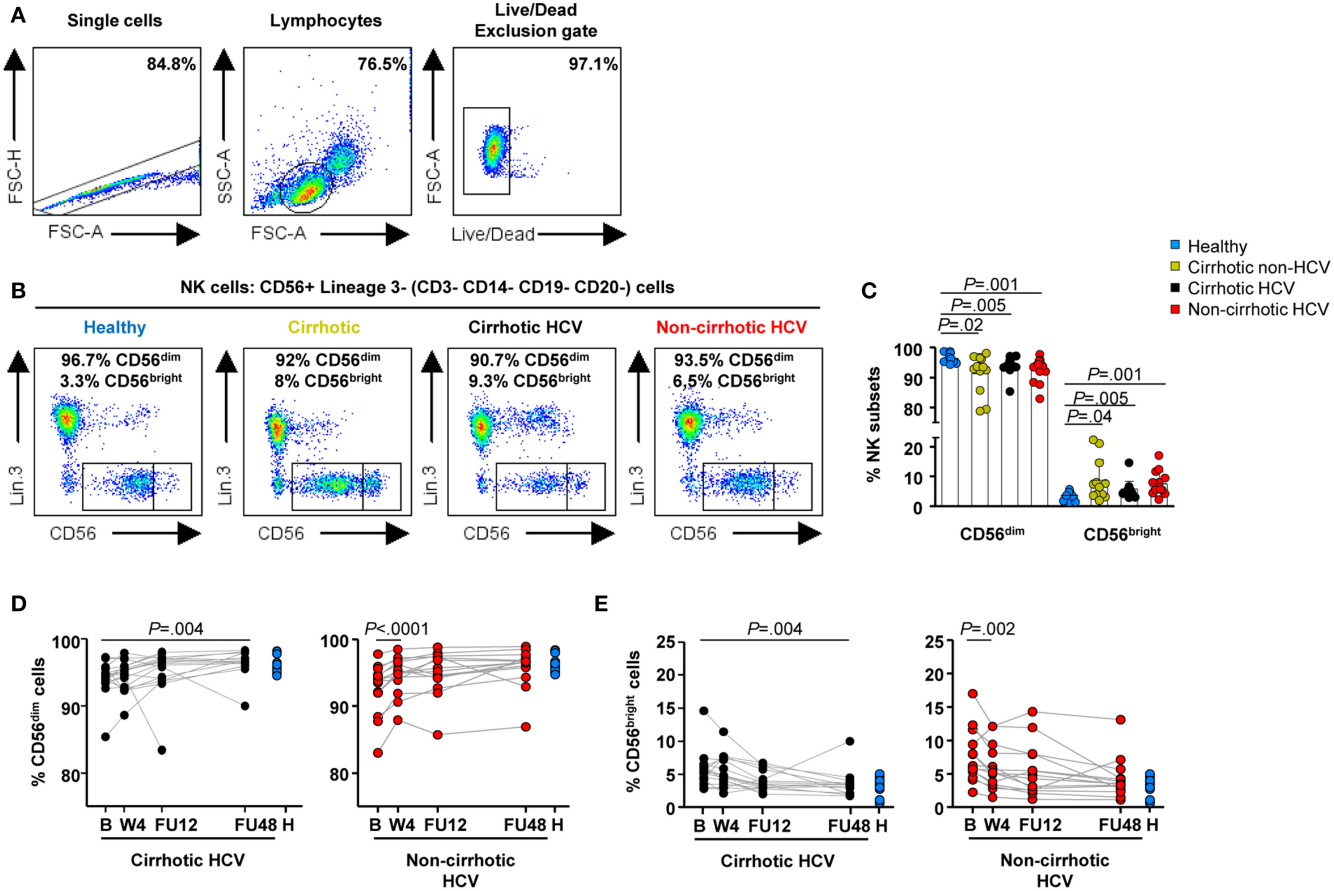
Delayed normalization of frequencies of NK subpopulations in HCV patients with cirrhosis. Flow cytometric analysis of PBMC samples from 12 healthy individuals (blue), 12 non-HCV cirrhotic controls (yellow), 17 HCV cirrhotic (black), and 14 non-cirrhotic (red) patients. **(A)** Representative sequential gating strategy used to identify NK cells, followed by representative flow cytometry dot blots **(B)** depicting NK subpopulations based on the expression of CD56 (CD56^dim^ and CD56^bright^ cells). **(C)** Baseline frequencies of CD56^dim^ (left) and CD56^bright^ cells (right). Error bars indicate mean + SD; *P*-values were determined by Mann–Whitney *U* test. Longitudinal analysis during and after IFN-free therapies in HCV cirrhotic and non-cirrhotic patients of CD56^dim^ cells **(D)** and CD56^bright^ **(E)** cells. *P*-values were determined by using mixed models for repeated measurements.

All 31 HCV-infected patients experienced a marked decay of serum HCV-RNA levels within the first 4 weeks of therapy {median [interquartile range (IQR)] decay: 5.96 [5.5–6.5], *P* < 0.0001} (Supplementary Figure 1A), along with normalization of liver tests (Supplementary Figures 1B–D). To investigate the impact of the DAA-mediated rapid elimination of HCV and the potential role of liver cirrhosis on NK cell phenotype and function restoration, we first analyzed circulating NK subpopulation frequencies (CD56^dim^ and CD56^bright^ subsets) and their receptor profile on the basis of the frequencies and expression levels of activating (HLA-DR, NKp46, and NKp30) and inhibitory (KIR2DL2/L3, NKG2A, and CD85j) receptors. NK cells were identified as CD3^−^ CD14^−^ CD19^−^ CD20^−^ CD56^+^ cells in the live lymphocyte population by sequential gating (Figures 1A,B).

Table 2 provides a concise summary of the time points at which normalization of NK phenotypic marker frequencies occurs for both HCV cirrhotic and non-cirrhotic patients. In detail, altered baseline frequencies of NK subpopulations were present in both HCV patients with and without cirrhosis, as well as in non-HCV cirrhotic controls compared with healthy controls (Figure 1C). These subset alterations normalized early on-therapy in non-cirrhotic patients (W4, *P* < 0.0001 and *P* = 0.002 for CD56^dim^ and CD56^bright^, respectively) (Figures 1D,E, right graphs), whereas in cirrhotics, their normalization was significantly delayed (FU48, *P* = 0.004 for both NK subsets) (Figures 1D,E, left graphs). No significant differences were observed in the percentage of total NK cells between study groups (Supplementary Figure 2).

**Table 2:** Summary of the normalization time points of NK cell frequency subsets and phenotype in cirrhotic and non-cirrhotic HCV patients.

		**Cirrhotic HCV**				**Non-cirrhotic HCV**			
		**CD56**^****dim****^		**CD56**^****bright****^		**CD56**^****dim****^		**CD56**^****bright****^	
		**Normalization**	***P***	**Normalization**	***P***	**Normalization**	***P***	**Normalization**	***P***
Subset frequency		FU48	0.004	FU48	0.004	W4	<0.0001	W4	0.002
Activating receptors	HLA-DR	FU48	<0.0001	FU48	0.001	FU48	0.0006	B	–
	NKp46	FU48	0.0002	FU48	<0.0001	W4	0.004	B	–
	NKp30	B	–	FU48	0.0007	B	–	FU48	<0.0001
Inhibitory receptors	NKG2A	FU48	n.s.	FU48	n.s.	B	–	B	–
	CD85j	^†^		n.a.		B	–	n.a.	
	KIR2DL2/L3	FU48	0.003	FU48	0.008	FU48	<0.0001	FU48	0.02

P-values correspond to the mixed models for repeated measurement analysis between baseline and the normalization time point. n.a., not applicable; n.s., not significant;

† *, not normalized at FU48; B, baseline (before therapy); W4, week 4 during therapy; FU12, 12 weeks after the end of therapy; FU48, 48 weeks after the end of therapy; NK, natural killer; HCV, hepatitis C virus*.

**Figure 2:**
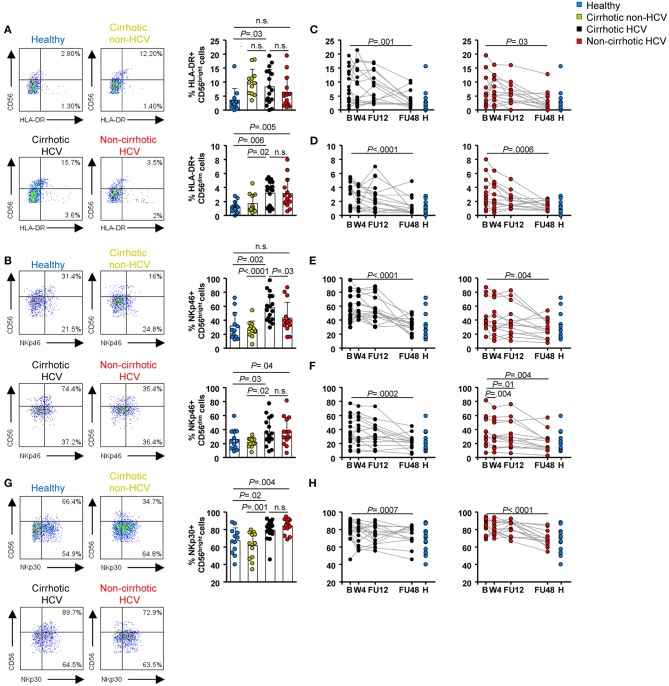
Activating receptor frequencies within NK subpopulations were upregulated at baseline in HCV patients with cirrhosis and persisted until FU48. Flow cytometric analysis of PBMC samples from 12 healthy individuals (blue), 12 non-HCV cirrhotic controls (yellow), 17 HCV cirrhotic (black), and 14 non-cirrhotic (red) patients. Representative flow cytometry plots and baseline frequencies of HLA-DR^+^ **(A)**, NKp46^+^ **(B)**, and NKp30^+^ **(G)** CD56^bright^ (above) and CD56^dim^ (below) cells. Longitudinal analysis during and after IFN-free therapies of HLA-DR^+^ CD56^bright^ cells **(C)**, HLA-DR^+^ CD56^dim^ cells **(D)**, NKp46^+^ CD56^bright^ cells **(E)**, NKp46^+^ CD56^dim^ cells **(F)**, and NKp30^+^ CD56^bright^ cells **(H)**. Error bars indicate mean + SD; *P*-values at baseline were determined by Mann–Whitney *U* test; *P*-values during longitudinal analysis were determined by mixed models for repeated measurements. n.s., not significant.

Frequencies of the activation marker HLA-DR and the NKp46 receptor were upregulated at baseline in HCV patients with cirrhosis compared with healthy controls within CD56^bright^ (*P* = 0.03 and *P* = 0.002, respectively) and CD56^dim^ (*P* = 0.006 and *P* = 0.03, respectively) subsets (Figures 2A,B). In addition, the frequency of HLA-DR^+^ CD56^bright^ cells was similar in both cirrhotic groups (HCV and non-HCV). These frequencies were not normalized earlier than FU48 in HCV cirrhotic patients (Figures 2C–F, left graphs). However, in HCV patients without cirrhosis, baseline frequencies of HLA-DR and NKp46 in CD56^bright^ cells did not differ from those in healthy controls (Figures 2C,E, right graphs). Additionally in this patient group, although baseline frequencies of these receptors in CD56^dim^ cells were higher than those in healthy controls (*P* = 0.005 and *P* = 0.04, respectively), they normalized rapidly at W4 for NKp46^+^ (*P* = 0.004) (Figure 2F, right graph) and at FU48 (*P* = 0.0006) for HLA-DR+ cells (Figure 2D, right graph). Finally, NKp30 frequencies at baseline in CD56^bright^ cells were also upregulated compared with those in healthy controls in HCV patients with (*P* = 0.02) or without cirrhosis (*P* = 0.004) (Figure 2G), and they normalized at FU48 in both patient groups (*P* = 0.0007 and *P* < 0.0001) (Figure 2H). This marker was also assessed in CD56^dim^ cells, but its expression in both patient groups did not differ at any time point from that in healthy controls (not shown).

**Figure 3:**
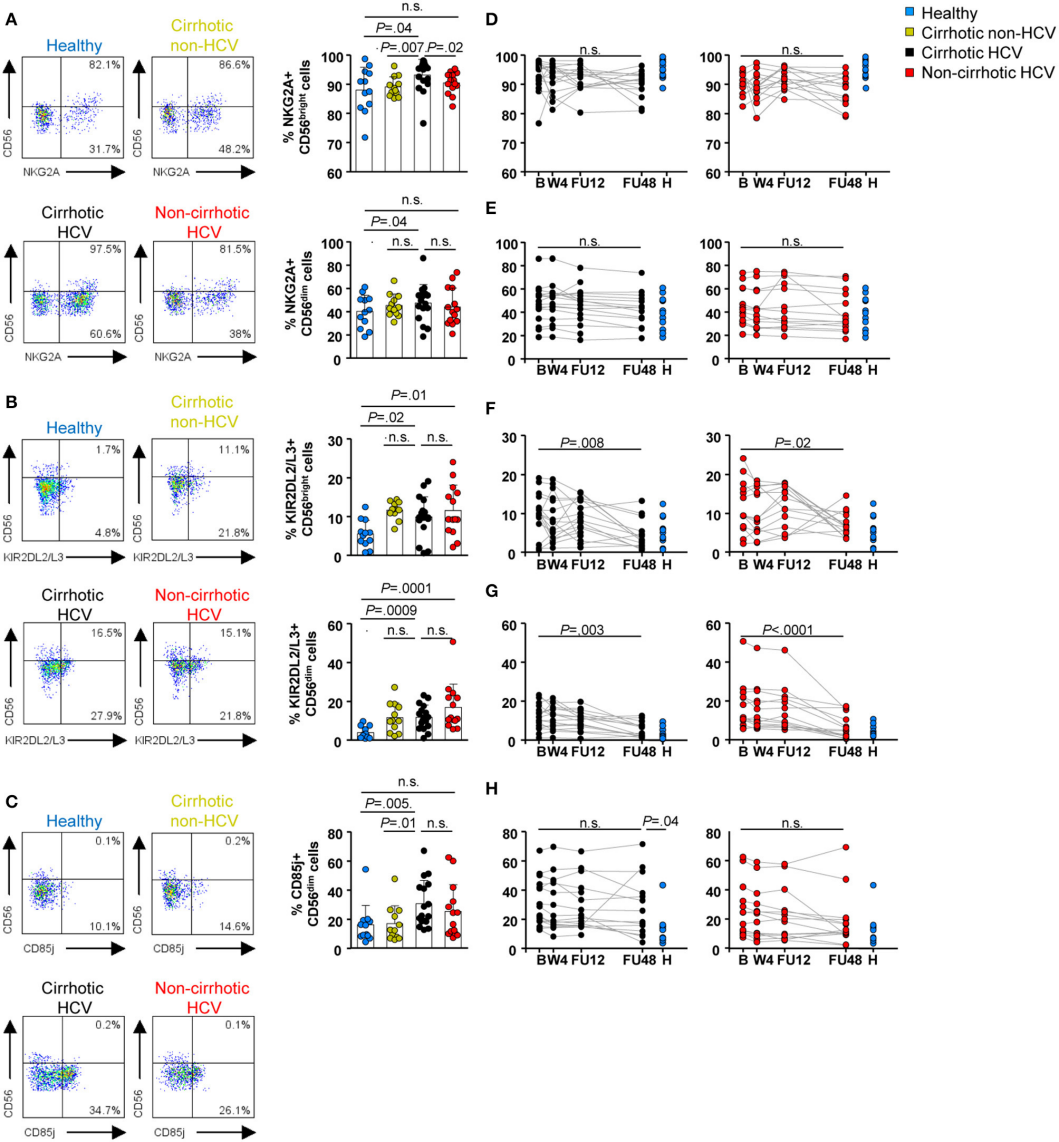
Inhibitory receptor frequencies within NK subpopulations were also upregulated at baseline in HCV patients with cirrhosis and persisted until FU48. Flow cytometric analysis of PBMC samples from 12 healthy individuals (blue), 12 non-HCV cirrhotic controls (yellow), 17 HCV cirrhotic (black), and 14 non-cirrhotic (red) patients. Representative flow cytometry plots and baseline frequencies of NKG2A^+^ **(A)**, KIR2DL2/L3^+^ **(B)**, and CD85j^+^ **(C)** CD56^bright^ (above) and CD56^dim^ (below) cells. Longitudinal analysis during and after IFN-free therapies of NKG2A^+^ CD56^bright^ cells **(D)**, NKG2A^+^ CD56^dim^ cells **(E)**, KIR2DL2/L3^+^ CD56^bright^ cells **(F)**, KIR2DL2/L3^+^ CD56^dim^ cells **(G)**, and CD85j^+^ CD56^dim^ cells **(H)**. Error bars indicate mean + SD; *P*-values at baseline were determined by Mann–Whitney *U* test; *P*-values during longitudinal analysis were determined by mixed models for repeated measurements. n.s., not significant.

Selected inhibitory receptors were also assessed. In HCV patients with cirrhosis, we detected elevated baseline frequencies of selected inhibitory receptors, with increased NKG2A^+^ CD56^dim^ and CD56^bright^ cells (*P* = 0.04 for both NK subsets) (Figure 3A), KIRDL2/L3^+^ CD56^dim^ (*P* = 0.0009) and CD56^bright^ (*P* = 0.02) cells (Figure 3B), and CD85j^+^ CD56^dim^ cells (*P* = 0.005) (Figure 3C). The CD85j receptor was not expressed on the CD56^bright^ subset, or its expression level was below the limit of the detection method (Figure 3C). Frequencies of NKG2A^+^ CD56^dim^ and KIR2DL2/L3^+^ CD56^dim^ and CD56^bright^ cells were similar between both cirrhotic groups (HCV and non-HCV). Again, normalization of inhibitory receptor profiles in both NK subsets in this patient group did not occur until FU48 (Figures 3D–G, left graphs), whereas CD85j expression remained elevated (Figure 3H, left graph). Notably, in the group of HCV patients without cirrhosis, baseline frequencies of NKG2A and CD85j cells were similar to those measured in healthy controls (Figures 3A,C). However, KIR2DL2/L3^+^ CD56^dim^ and CD56^bright^ cells were elevated compared with those in healthy controls (*P* = 0.007 and *P* = 0.04, respectively) (Figure 3B), but they also normalized at FU48 (*P* < 0.0001 and *P* = 0.02, respectively) (Figures 3F,G, right graphs).

Receptor expression, as deduced by the mean fluorescence intensity (MFI), was also assessed. At baseline, elevated expression of NKp30, and NKp46 receptors within the CD56^bright^ subset was detected as well as for NKG2A within both CD56^dim^ and CD56^bright^ cells in HCV patients with and without cirrhosis compared with healthy and non-HCV cirrhotic controls (Supplementary Figure 3). In HCV patients with cirrhosis, similarly to the delayed restoration of the frequencies at FU48 of NK subset and markers, MFI of NKp30, NKp46, and NKG2A normalized at the same time point (Supplementary Figures 4A–D, left graphs). In contrast, in non-cirrhotic HCV patients, NKp46, and NKG2A MFI of CD56^bright^ cells were normalized earlier (at W4 and FU12, respectively) (Supplementary Figures 4B,C, right graphs).

### Natural Killer Cell Function Alterations and Their Restoration Kinetics Are Associated With Cirrhosis in Hepatitis C Virus Patients

**Figure 4:**
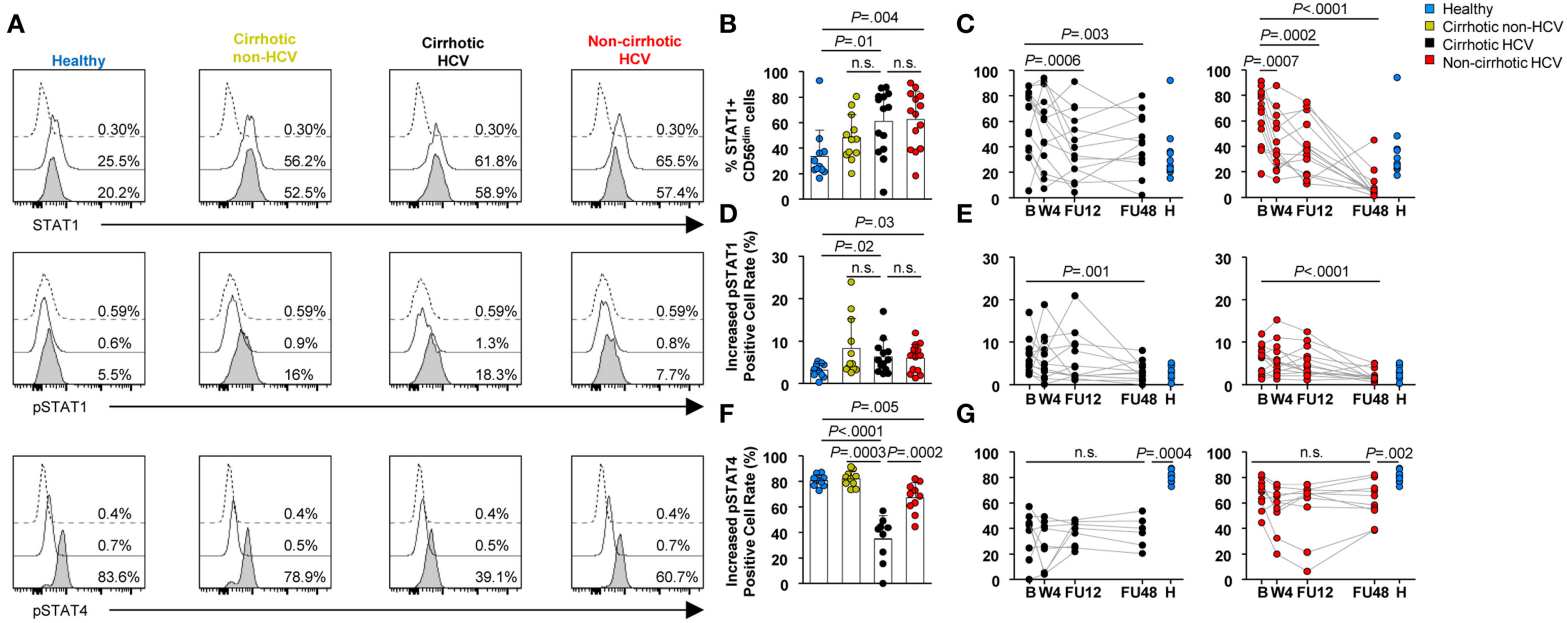
Profound alterations in STAT1/4 activation in response to IFN-α in CD56^dim^ cells from HCV patients with cirrhosis. **(A)** Representative histograms of a healthy subject, non-HCV cirrhotic control, HCV cirrhotic, and non-cirrhotic patients are shown. Dotted lines show staining of cells with isotype control; thick lines show staining of unstimulated cells; and thick lines with shaded areas show staining of IFN-stimulated cells. Comparison of baseline frequencies and longitudinal analysis of STAT1^+^ CD56^dim^ cells **(B,C)**, pSTAT1 **(D,E)**, and pSTAT4 **(F,G)** levels in response to IFN-α between healthy controls, non-HCV cirrhotic controls, cirrhotic HCV, and non-cirrhotic HCV patients. Increased positive cell rates were determined by subtracting the positive cell rate of unstimulated cells from that of stimulated cells. Error bars indicate mean + SD; *P*-values at baseline were determined by Mann–Whitney *U* test; *P*-values during longitudinal analysis were determined by mixed models for repeated measurements. n.s., not significant.

To assess whether HCV and established liver cirrhosis would have an impact on the capacity of NK cells to perform effector functions, we first assessed their response to type I IFN. Specifically, STAT1, pSTAT1, and pSTAT4 levels were quantified post-IFN-α stimulation *in vitro* in CD56^dim^ cells, because they comprise the most cytotoxic population (22). Frequency and expression level (MFI) of STAT1^+^ cells at baseline were significantly increased in patients with HCV infection regardless of their cirrhosis status compared with that in healthy controls and in patients with cirrhosis of other etiologies (*P* ≤ 0.01) (Figures 4A,B and Supplementary Figure 5A). These levels normalized at FU12 in HCV cirrhotic patients (*P* = 0.0006 for the frequency and *P* = 0.001 for the MFI), whereas frequency and MFI of STAT1^+^ cells normalized at W4 in non-cirrhotics (*P* = 0.0007 and *P* = 0.03, respectively) (Figure 4C and Supplementary Figures 5B–C). Furthermore, in response to IFN-α, the phosphorylation level of STAT1 was also elevated in all HCV patients and in non-HCV cirrhotic controls compared with healthy subjects (Figures 4A,D). Again, pSTAT1^+^ level normalized earlier in HCV non-cirrhotics than in HCV cirrhotics (FU12 vs. FU48), although statistically significant differences were assessed at FU48 (Figure 4E). In contrast, stimulation with IFN-α led to a weaker STAT4 phosphorylation in CD56^dim^ cells from HCV patients than in those from healthy subjects or non-HCV cirrhotic controls (Figures 4A,F). Importantly, pSTAT4 level was significantly higher in HCV without cirrhosis than in HCV cirrhotics (*P* = 0.0002), although it remained significantly lower compared with that in healthy controls at FU48 in both HCV groups (*P* = 0.0004 for cirrhotics and *P* = 0.002 for non-cirrhotics) (Figure 4G).

**Figure 5:**
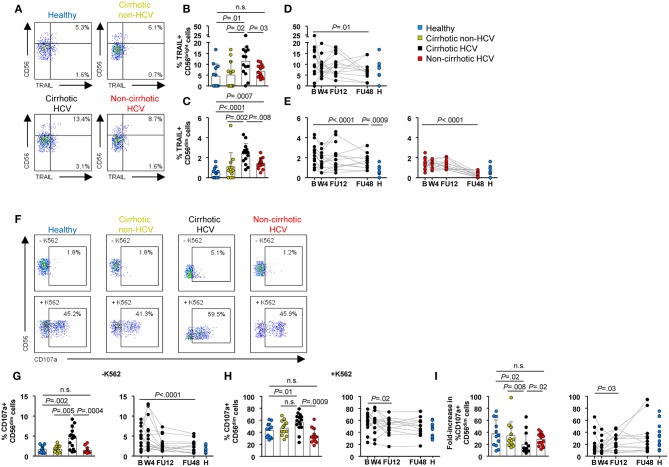
NK cytotoxicity differed between HCV patients with and without cirrhosis at baseline and remained upregulated in cirrhotics after IFN-free therapies. **(A)** Representative flow cytometry plots, **(B,C)** baseline frequencies, and **(D,E)** longitudinal analysis of TRAIL within CD56^bright^ and CD56^dim^ cells. **(F)** Representative flow cytometry plots of CD107a^+^ CD56^dim^ cell frequencies with or without coincubation with K562. **(G)** *Ex vivo* baseline frequency of CD107a^+^ CD56^dim^ cells and longitudinal analysis. **(H)** Baseline frequency of CD107a^+^ CD56^dim^ cells and longitudinal analysis upon stimulation with K562 cells. **(I)** Degranulation capacity (fold increase) of CD56^dim^ cells at baseline and during longitudinal analysis. PBMC samples from 14 cirrhotic and 15 non-cirrhotic HCV patients were available in sufficient cell numbers for the evaluation of TRAIL and CD107a expression, respectively.

Next, we assessed NK cell cytotoxic capacity, which could be exerted either by direct killing of target cells through death ligands, such as TRAIL or release of lytic granules [surface CD107a expression (23)]. Because TRAIL expression is greater in CD56^bright^ than in CD56^dim^ cells, we also gated on this subpopulation (24). Baseline frequencies of TRAIL within CD56^bright^ and CD56^dim^ subpopulations were higher in HCV cirrhotic patients compared with healthy (*P* = 0.01 and *P* < 0.0001, respectively) and non-HCV cirrhotic control groups (*P* = 0.02 and *P* = 0.002, respectively) (Figures 5A–C). In HCV patients without cirrhosis, the frequency of TRAIL^+^ CD56^bright^ cells at baseline was similar to that in healthy controls and significantly lower than that in HCV patients with cirrhosis (*P* = 0.03) (Figure 5B). In contrast, the frequency of TRAIL within CD56^dim^ cells was higher than that in healthy controls (*P* = 0.0007), but it was significantly lower than that in HCV cirrhotic patients (*P* = 0.008) (Figure 5C).

Interestingly, we also observed a correlation between liver stiffness values, measured by transient elastography (FS) and frequencies of TRAIL within CD56^dim^ and CD56^bright^ NK subsets (*R*^2^ = 0.51 *P* = 0.007 for CD56^dim^ cells and *R*^2^ = 0.53, *P* = 0.005 for CD56^bright^ cells) (Supplementary Figure 6). Moreover, because TRAIL itself is an IFN-stimulated gene (ISG), whose expression depends on the STAT1/pSTAT1 pathway (25), the link between STAT1/pSTAT1 and TRAIL expression is underscored in HCV cirrhotic patients by the correlation between the frequencies of TRAIL^+^ and either STAT1^+^ or pSTAT1^+^ cells within the CD56^dim^ subset (*R*^2^ = 0.61, *P* = 0.04 and *R*^2^ = 0.70, *P* = 0.02, respectively) (Supplementary Figure 7).

Of note, there was a significant decrease in the frequency of TRAIL^+^ CD56^dim^ cells at FU48 in both HCV cirrhotic and non-cirrhotic patients (*P* < 0.0001 for both groups), albeit only patients without cirrhosis achieved normal levels comparable with those of healthy controls. On the other hand, in patients with HCV cirrhosis, the frequency of TRAIL^+^ CD56^bright^ cells normalized at FU48 (*P* = 0.01) (Figures 5D,E).

To shed more light into the distinct normalization kinetics of the frequencies of STAT1/pSTAT1, as well as into the higher increased pSTAT4 positive cell rate and lower baseline frequencies of TRAIL, in non-cirrhotic vs. cirrhotic HCV patients, serum IFN-α levels were measured and compared with those of the control groups. IFN-α protein showed a non-significant trend to be at higher concentrations in HCV patients with cirrhosis compared with those without cirrhosis, showing only a slow and partial decrease on- and post-therapy (Supplementary Figure 8). However, IFN-α was detectable at high levels only in a minority of patients (3/17).

*Ex vivo* degranulating CD107a^+^ CD56^dim^ cells were also found at an increased frequency at baseline in HCV patients with cirrhosis compared with healthy (*P* = 0.002) and non-HCV cirrhotics (*P* = 0.005) (Figures 5F,G) and once again normalized by FU48 (*P* < 0.0001) (Figure 5G, right graph). Of note, the frequency of degranulating NK cells in HCV patients without cirrhosis did not differ from that in healthy controls. After K562 coincubation, CD107a expression in CD56^dim^ cells was higher in HCV cirrhotics compared with healthy (*P* = 0.01) and non-cirrhotics (*P* = 0.0009) (Figure 5H, left graph). Nevertheless, the degranulation response (fold change) induced upon K562 cell stimulation was significantly lower in HCV cirrhotics compared with healthy (*P* = 0.02), non-HCV cirrhotic (*P* = 0.008), and non-cirrhotics (*P* = 0.02) (Figure 5I, left graph). Longitudinal analysis in HCV cirrhotics showed that this response normalized at FU12 (Figures 5H,I, right graphs).

Collectively, these data indicate that non-cirrhotic HCV patients have a NK cell profile that more closely resembles or more rapidly returns to that of healthy donors, whereas NK cell normalization in cirrhotic HCV patients is incomplete and late post-therapy.

### Natural Killer Cell Cytokine Production Could Be Restored in Non-cirrhotics While Remaining Totally Impaired in Cirrhotic Hepatitis C Virus Patients

An essential function of NK cells, especially in viral infections, is to release cytokines, such as IFN-γ and tumor necrosis factor-α (TNF-α) as immune-defensive agents (26). Therefore, the ability of CD56^dim^ and CD56^bright^ NK subsets to produce cytokines was evaluated. In a first set of experiments, cytokine production was assessed after *in vitro* stimulation with IL-12 and IL-15. By this conventional method, mostly CD56^bright^ cells were found to produce IFN-γ and TNF-α, whereas cytokine-producing CD56^dim^ cells were below 15% in the majority of individuals, in agreement with data obtained in previous studies (9). CD56^dim^ cells show real IFN-γ production at early time intervals after cytokine stimulation (0–16 h) (27). Nevertheless, we could not detect intense and accumulating IFN-γ production by CD56^dim^ cells with this approach because PBMCs were cryopreserved until the moment of analysis (data not shown). For this reason, to allow detection of cytokine production in the CD56^dim^ subset, we incubated PBMCs with IL-12 and IL-15 in the presence of K562 target cells, as previously described (28).

**Figure 6:**
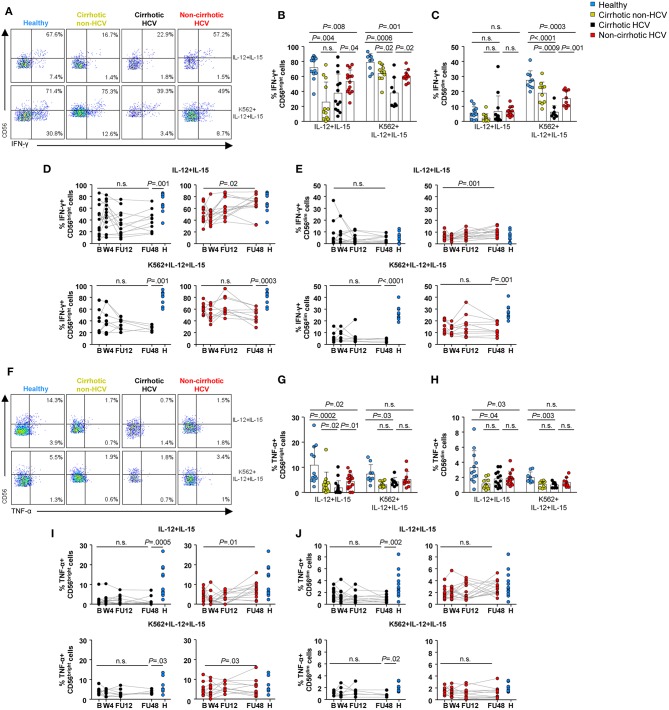
NK cell cytokine production was not restored in HCV patients with cirrhosis 1 year after the end-of-therapy. Representative flow cytometry plots **(A)** and baseline frequencies of IFN-γ^+^ CD56^bright^ **(B)** and CD56^dim^ **(C)** cells after IL-12 and IL-15 or IL-12, IL-15, and K562 stimulation. Longitudinal analysis of IFN-γ-producing CD56^bright^ **(D)** and CD56^dim^ **(E)** cells in cirrhotic and non-cirrhotic HCV patients. Representative flow cytometry plots **(F)** and baseline frequencies of TNF-α^+^ CD56^bright^ **(G)** and CD56^dim^ **(H)** cells after IL-12 and IL-15 or IL-12, IL-15, and K562 stimulation. Longitudinal analysis of TNF-α-producing CD56^bright^ **(I)** and CD56^dim^ **(J)** cells in cirrhotic and non-cirrhotic HCV patients. *P-*values were determined during the longitudinal analysis by using mixed models for repeated measurements, and *P*-values between healthy controls and the different time point were determined by Mann–Whitney *U* test; n.s., not significant.

At baseline, the frequency of IFN-γ-producing CD56^bright^ cells was significantly lower in HCV patients compared with healthy controls, regardless of the stimulation procedure (*P* < 0.01) (Figures 6A,B). However, this defect was less marked in HCV patients without cirrhosis. Of note, a similar low frequency was also assessed in non-HCV cirrhotic controls after IL-12 and IL-15 stimulation. Notable IFN-γ production in CD56^dim^ cells was detectable upon target-cell recognition with K562 cells. Similar to that observed in the CD56^bright^ subset, the frequency of IFN-γ^+^ CD56^dim^ cells was lower in HCV patients compared with healthy subjects (*P* < 0.0001 in HCV cirrhotics and *P* = 0.003 in non-cirrhotics), even though it was significantly higher in patients without cirrhosis than in those with cirrhosis (*P* = 0.001) (Figures 6A,C). During follow-up, the frequencies of IFN-γ-producing NK subsets upon exposure to IL-12, IL-15, and/or K562 cells in HCV cirrhotic patients remained at low levels and did not normalize. On the contrary, the frequency of IFN-γ^+^ CD56^bright^ cells after *in vitro* stimulation with IL-12 and IL-15 normalized at FU48 in non-cirrhotics (*P* = 0.02) (Figures 6D,E).

The baseline frequencies of TNF-α-producing CD56^dim^ and CD56^bright^ cells were significantly decreased in HCV cirrhotic patients compared with healthy controls with or without K562 cells (Figures 6F–H). After cytokine exposure (IL12 and IL-15), these frequencies were also lower in HCV non-cirrhotic patients compared with healthy subjects (*P* = 0.02 for CD56^bright^ and *P* = 0.03 for CD56^dim^), although they normalized at FU48. Once again, the frequencies of TNF-α within either CD56^bright^ or CD56^dim^ cells in HCV cirrhotic patients did not reach similar levels to those observed in healthy controls even up to FU48 (Figures 6I,J).

Finally, it is important to point out that we did not observe any statistically significant differences in either the expression of NK cell markers or NK cell functions between previous IFN-α treated or naïve patients in either the cirrhotic or non-cirrhotic HCV groups (Supplementary Figure 9). In addition, we did not find any statistically significant differences between control patients with alcoholic or NASH-related cirrhosis (Supplementary Figure 10).

## Discussion

This study describes, to the best of our knowledge, the first comprehensive comparison of the phenotype and function of NK subpopulations throughout a longitudinal analysis between HCV-infected patients with or without cirrhosis undergoing IFN-free therapies. Previous findings presented imbalanced NK subpopulation frequencies at baseline (29), accompanied by high expression of the activating receptors NKp46 (30), NKp30 (31), and HLA-DR (11) and the inhibitory receptors KIR2DL2/L3 (32), NKG2A (33), and CD85j (11) in HCV-infected patients. However, our cross-sectional analysis revealed marked differences in activating and inhibitory receptors of NK cells from patients with HCV-related cirrhosis and to a lesser extent in patients with cirrhosis of other etiologies compared with healthy controls. Although an imbalance of NK subpopulations was also observed in HCV non-cirrhotic patients, differences in NK receptor expression were only subtle. This striking difference in cirrhotic vs. non-cirrhotic patients may be the result of deregulated homeostatic mechanisms induced by long-term liver damage during cirrhosis. Because the liver is a major contributor to the homeostasis of the innate immune system (34), our results suggest that liver injury, together with chronic HCV infection, contributes to substantial alterations in NK cell phenotype. This could be further supported by the increased frequencies of HLA-DR, KIR2DL2/L3, and NKG2A in non-HCV cirrhotic controls. Moreover, in the longitudinal monitoring, we also observed differences in the restoration kinetics between cirrhotic and non-cirrhotic HCV patients, despite rapid viral eradication and normalization of liver tests in both groups. Overall, NK subpopulation frequencies and receptor expression were restored at FU48 in cirrhotics (except CD85j, discussed below), whereas a rapid restoration at W4 was observed in non-cirrhotic patients for NK cell frequencies and NKp46^+^ CD56^dim^ cells. Again, these results may be explained by the degree of liver disease and likely by an extended HCV infection time in individuals with liver cirrhosis.

Krämer et al. have shown that NKp46^high^ expression defines a specific NK cell subset that may be involved in the suppression of both HCV replication and HCV-associated liver damage (35). Our data are in line with these observations, because prior to DAA therapies, we also assessed NKp46^high^ expression at least in CD56^dim^ cells in all HCV patients, underpinning the role of NK cells in the immunopathogenesis of HCV. Moreover, in HCV patients with cirrhosis, NKp46^high^ expression (in both CD56^dim^ and CD56^bright^ subsets) normalized at FU48, further supporting the notion that NK46^high^-expressing NK cells play a role in modulating liver fibrosis.

Previous data have suggested that constant exposure to type I IFN during chronic HCV infection, which can be produced by a variety of cell types, contributes to the prolonged activation status of NK cells. Hence, IFN-α induces increased expression and phosphorylation of STAT1 over STAT4 and, consequently, induction of ISG and polarization of NK cell function toward cytotoxicity, as evidenced by increased degranulation and production of TRAIL (25). However, in our study, we detected elevated degranulation and TRAIL expression only in the cirrhotic group. Of note, IFN-α was hardly detected in serum of HCV patients as previously reported (36). Because we were not able to measure IFN-α level in biopsies, it remains possible that sustained induction of intrahepatic IFN-α, evidenced by elevated ISG expression (STAT1 and TRAIL) and degranulation in peripheral NK cells, may be a driver of prolonged NK cell abnormalities in HCV cirrhotic patients. Indeed, TRAIL levels ostensibly correlated to STAT1/pSTAT1 levels in this group.

In the healthy liver, hepatic stellate cells (HSCs) and subsequently hepatocytes are shielded from NK cells by endothelial cells. However, this protection is lost during liver damage (37), and thus, NK cells can eliminate hepatocytes in a TRAIL-mediated mechanism (38). Indeed, our results show at baseline a clear upregulation of TRAIL in CD56^dim^ cells, the most cytotoxic subset, in both HCV groups indicating that at baseline NK cells may play a role in the elimination of infected hepatocytes. Moreover, NK cells have been also implicated in fibrosis control via TRAIL-mediated killing of HSCs (39). In particular, it has been shown that NK cell-mediated induction of HSC apoptosis is an HCV-associated phenomenon, although NK cell-induced HSC apoptosis was inversely associated with liver fibrosis stage (40). In our study, we also observed an elevated frequency of TRAIL^+^ CD56^dim^ cells even at FU48 in HCV cirrhotic patients and a clear correlation between the baseline frequencies of TRAIL within both NK subsets and liver stiffness measurements. TRAIL-producing NK cells in patients with cirrhosis have been shown to kill hepatocytes in HCV/HBV infection (24, 41) and to actively promote necroinflammatory damage. Thus, TRAIL-producing NK cells may be a contributing factor to liver injury and fibrosis progression.

NK cell cytotoxic activity was assessed in our study *ex vivo* spontaneously or under co-culture with target K562 cells without cytokine addition. Spontaneous release of CD107a by NK cells was elevated only in HCV patients with cirrhosis. This result may reflect recirculation from tissue milieus where NK cells have been triggered extensively instead of an *in vivo* cytotoxic status. Although we were able to show an increase in the frequency of CD107a^+^ CD56^dim^ cells upon K562 cells co-cultivation (Figure 5H), the degranulation response (fold change) was significantly lower in HCV patients with cirrhosis compared with all other groups. These data suggest an impaired ability of NK cells from HCV patients with cirrhosis to be activated *ex vivo*, and it is restored at FU12. These results are in line with data published by Serti et al. demonstrating an impairment of NK cells to degranulate in response to IFN-α (12), which is restored upon DAA therapies. Our data clearly demonstrate that this impairment is associated with HCV infection and cirrhosis because HCV patients without cirrhosis and non-HCV cirrhotic controls presented similar fold increase in CD107a^+^ CD56^dim^ cells, compared with healthy individuals.

Consistent with previous studies (9, 10), our results show decreased baseline frequencies of either IFN-γ- or TNF-α-producing NK (CD56^dim^ and CD56^bright^) cells in patients with cirrhosis regardless of their HCV status. It is worth nothing that these defects were more marked in patients with cirrhosis. Because we analyzed the NK cell capacity to produce cytokines after IL-12 and IL-15 stimulation, these results demonstrate NK cell exhaustion in response to cytokines due to chronic HCV infection, which is exacerbated in patients with cirrhosis, as evidenced by the poor response to cytokine stimulation observed in cirrhotic patients from other etiologies (42). Importantly, this effector function normalized during the follow-up in HCV non-cirrhotic patients for the CD56^bright^ subset, whereas in cirrhotic patients, at least for the analyzed time frame, it remained altered. Although our stimulation method has a bias toward losing IFN-γ production by CD56^dim^ NK cells that produce it at early time intervals (0–16 h) (27), CD56^dim^ cells excel in cytokine production upon recognition of K562 target cells (28). Our results show that neither IL12 + IL15 nor K562 + IL12 + IL15 induced normal cytokine production by both CD56^dim^ and CD56^bright^ subsets in cirrhotic HCV patients at all time points, indicating that the ability of NK cells to produce IFN-γ and TNF-α is impaired in this patient group.

According to Miyagi et al. (43), our data indicate that elevated phosphorylation of STAT1 over STAT4 after IFN-α stimulation should result in induction of ISG expression (i.e., STAT1 and TRAIL), enhancement of degranulation, and poor IFN-γ production in HCV patients with cirrhosis. In addition, impaired cytokine production by NK cells in cirrhotics could be also partially explained by the elevated frequency of CD85j^+^ CD56^dim^ cells because it has been demonstrated that blocking of CD85j receptor increases the secretion of IFN-γ in NK cells (44).

Our study focused on the phenotypic and functional analysis of peripheral NK cells, raising the question of the origin of the changes described here. The liver is an organ rich in NK cells imprinted in a liver-specific manner. These intrahepatic NK cells contain liver resident NK cells, memory-like NK cells, and transient conventional NK cells represented mainly by recirculating CD56^dim^ cells through the liver blood system (45). However, in cirrhosis, normal liver architecture is replaced by nodules of hepatocytes surrounded by wide streets of fibrotic tissues, which massively constrict blood flow and reduce liver function (37). Thus, our data may reflect altered recirculation patterns of peripheral, lymphoid, or intrahepatic NK cells in HCV cirrhosis due to liver microcirculatory dysfunction. Because liver biopsies, which could have provided intrahepatic resident NK cells, are not routinely used in clinical practice any more, our study carries the limitation to analyze only peripheral NK cells.

Finally, we observed that phenotypic and functional NK cell restoration dynamics follow a distinct pattern in HCV patients with cirrhosis: whereas activating and inhibitory receptor expression (except CD85j) normalizes at FU48, most of the functional markers remain altered even at that late time point. It has been described that activating receptor signaling promotes degranulation and cytokine production in NK cells under steady-state conditions (46–49). Although in our study we observed upregulated frequencies of NKp46, NKp30, and HLA-DR, we did not observe any significant correlation between the progressive normalization of receptor expression with the degranulation marker (CD107a) or with cytokine production (IFN-γ and TNF-α) (data not shown). Rather, our results of higher expression of STAT1/pSTAT1 and CD107a expression accompanied by lower pSTAT4 expression and cytokine production at baseline in HCV patients with cirrhosis are in line with data published by Ahlenstiel et al. describing a polarized NK cell phenotype toward cytotoxicity away from IFN-γ production (9).

As mentioned above, the damaged liver structure in cirrhosis, which leads to systemic inflammation and immune dysfunction, may cause distinct receptor alterations unlinked to the functional NK cell dynamics. Interestingly, fibrosis and even cirrhosis regression in decompensated patients have been described post-SVR, albeit this process is long-lasting (50) and thus ultimately may compromise NK functional recovery. Further studies will decipher the complex NK cell signaling pathway, which involves several activating and inhibitory receptors, and how these are interconnected to NK cell functions and finally the long-term impact on NK cells in the context of HCV-related cirrhosis.

In conclusion, we provide compelling evidence that, unlike non-cirrhotic patients, NK cells in HCV patients with cirrhosis present a delayed phenotype normalization and incomplete functional recovery after successful all-oral antiviral therapy. This altered immunosurveillance may have significant implications, considering the prominent role that NK cells play in fibrosis progression and hepatocellular carcinoma (HCC) development, and this deserves further clinical attention.

## Data Availability Statement

All datasets generated for this study are included in the article/Supplementary Material.

## Ethics Statement

The studies involving human participants were reviewed and approved by Comité Ético de Investigación Clínica del Hospital Clínic (Barcelona). The patients/participants provided their written informed consent to participate in this study.

## Author Contributions

EPe contributed to the study design, performed and analyzed experiments, and wrote the manuscript. SP-D-P and M-CL contributed to the study design and data interpretation. M-CL, ZM, EPo, and SL contributed to the patient recruitment and cohort follow-up. CB, PG, and MG-L provided the technical assistance. MM provided the critical assistance in study design and data interpretation. XF and GK contributed to the study design and supervision and wrote the manuscript. All authors have contributed to the critical revision of the manuscript and given final approval of the published version, including the authorship list.

### Conflict of Interest

XF has acted as advisor for Gilead and Abbvie, and has received unrestricted grant support from Abbvie. MM has received unrestricted grant support from Gilead, Roche, and Immunocore and has sat on advisory boards for Gilead, Roche, Arbutus, and Janssen. The remaining authors declare that the research was conducted in the absence of any commercial or financial relationships that could be construed as a potential conflict of interest.

## Abbreviations

ALT: alanine aminotransferase
AST: aspartate aminotransferase
B: baseline
DAA: direct-acting antiviral
DCV: daclatasvir
DSV: dasabuvir
EBR: elbasvir
FIB-4: fibrosis-4 score
FS: FibroScan
FU12: 12 weeks after end of therapy
FU48: 48 weeks after end of therapy
GGT: gamma-glutamyl transferase
GZR: grazoprevir
HCC: hepatocellular carcinoma
HCV: hepatitis C virus
HSC: hepatic stellate cell
IFN: interferon
IL-12: interleukin 12
IL-15: interleukin 15
ISG: interferon-stimulated gene
LDV: ledipasvir
MELD: model for End-stage liver disease
MFI: mean fluorescent intensity
NK: natural killer
PBMC: peripheral blood mononuclear cell
pSTAT1: phosphorylated signal transducer and activator of transcription 1-alpha/beta
pSTAT4: phosphorylated signal transducer and activator of transcription 4-alpha/beta
PTV/r/OBV: paritaprevir/ritonavir/ombitasvir
RBV: ribavirin
SOF: sofosbuvir
STAT1: signal transducer and activator of transcription 1-alpha/beta
TNF-α: tumor necrosis factor-α
TRAIL: tumor necrosis factor-related apoptosis-inducing ligand
VEL: velpatasvir
W4: week 4
WBC: white blood cells.
