# Supplementary material for: Cirrhosis Hampers Early and Rapid Normalization of Natural Killer Cell Phenotype and Function in Hepatitis C Patients Undergoing Interferon-Free Therapy
Source: Front Immunol. 2020 Feb 25;11:129. doi: 10.3389/fimmu.2020.00129 (PMC7052355; doi:10.3389/fimmu.2020.00129)
Supplement: Supplementary file 1 [file Data_Sheet_1.docx]

**SUPPLEMENTARY INFORMATION:**

Cirrhosis Hampers Early and Rapid Normalization of Natural Killer Cell Phenotype and Function in Hepatitis C Patients Undergoing Interferon-Free Therapy.

Elena Perpiñán, Sofía Pérez-Del-Pulgar, María-Carlota Londoño, Zoe Mariño, Sabela Lens, Concepción Bartres, Patricia González, Mireia García-López, Elisa Pose, Mala K. Maini, Xavier Forns, George Koutsoudakis

[Supplementary Figures 2](#_Toc25076433)

[Sl. Fig. 1 2](#_Toc25076434)

[Sl. Fig. 3 4](#_Toc25076435)

[Sl. Fig. 4 5](#_Toc25076436)

[Sl. Fig. 5 6](#_Toc25076437)

[Sl. Fig. 6 7](#_Toc25076438)

[Sl. Fig 8 9](#_Toc25076439)

[Sl. Fig. 9 10](#_Toc25076440)

[Sl. Fig. 10 11](#_Toc25076441)

[Supplementary Tables 12](#_Toc25076442)

[Sl. Table 1 12](#_Toc25076443)

# Supplementary Figures

## Sl. Fig. 1


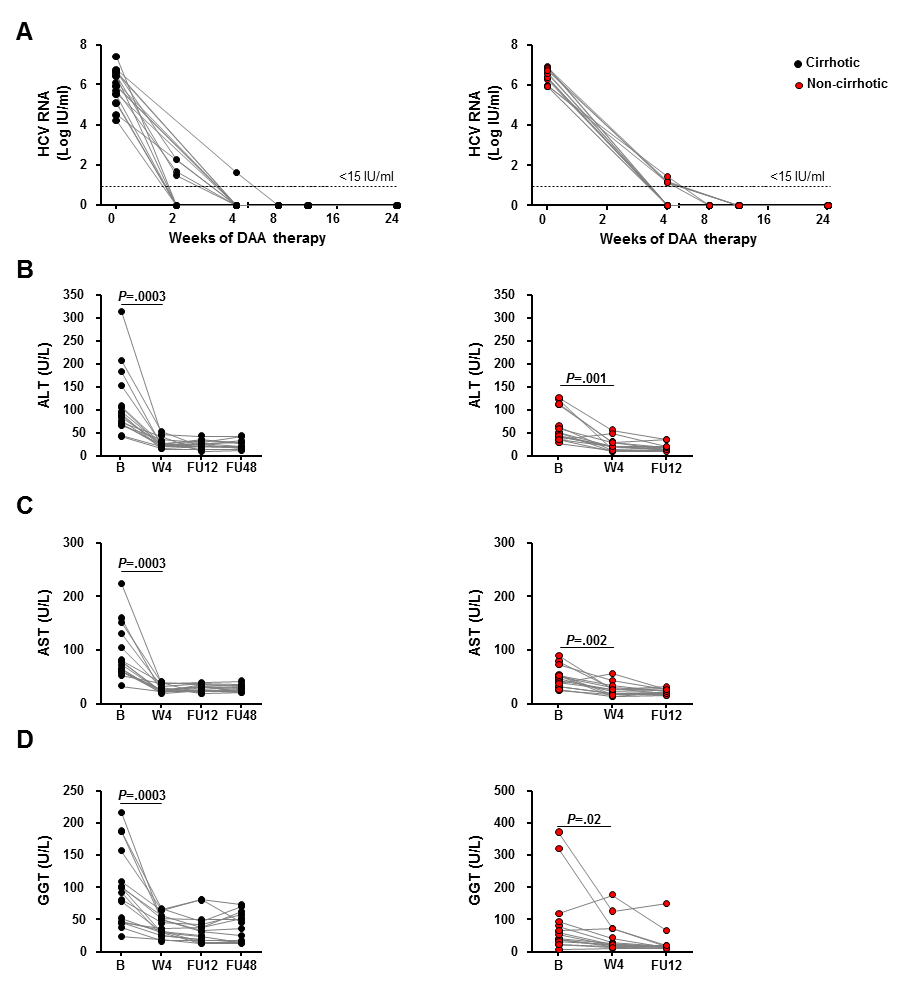


**Supplementary Figure 1. Rapid decay of HCV viremia and normalization of liver inflammation markers during IFN-free therapy.** (A) Serum HCV-RNA, (B) ALT (alanine aminotransferase), (C) AST (aspartate aminotransferase) and (D) GGT (gamma-glutamyl transferase) decay in 17 HCV cirrhotic (black, right graphs) and 14 non-cirrhotic (red, left graphs) patients during and after IFN-free therapy. P-values were determined by Wilcoxon signed-rank test. B, baseline; W4, week 4 during therapy; FU12, 12 weeks after the end-of-therapy; FU48, 48 weeks after the end-of-therapy.

**Sl. Fig. 2**

**
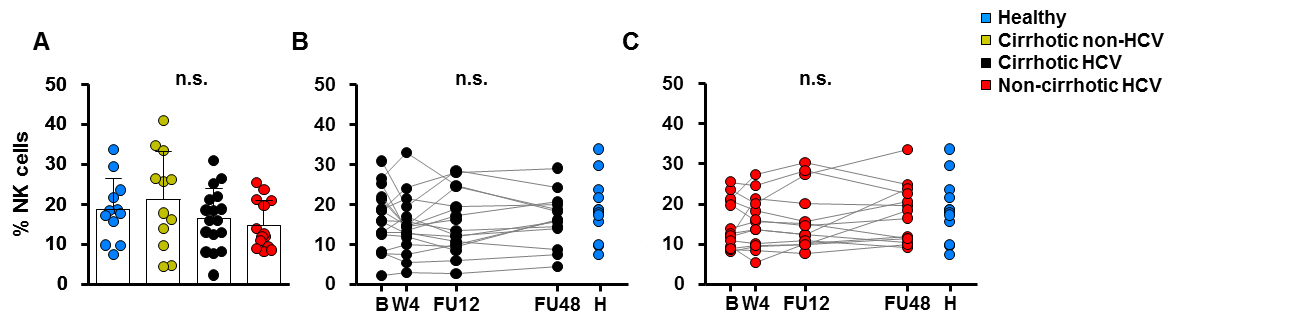
**

**Supplementary Figure 2. Frequency of total NK cells in HCV cirrhotic and non-cirrhotic patients.** (A) Baseline frequencies of total NK cells in 12 healthy individuals, 12 non-HCV cirrhotic patients, 17 HCV cirrhotic and 14 non-cirrhotic patients. Error bars indicate mean ±SD; p-values were calculated by Mann-Whitney U test. Longitudinal analysis of the frequency of total NK cells during and after IFN-free therapies in (B) HCV cirrhotic and (C) non-cirrhotic patients. P-values were determined by using Mixed Models for Repeated Measurements. .

## Sl. Fig. 3


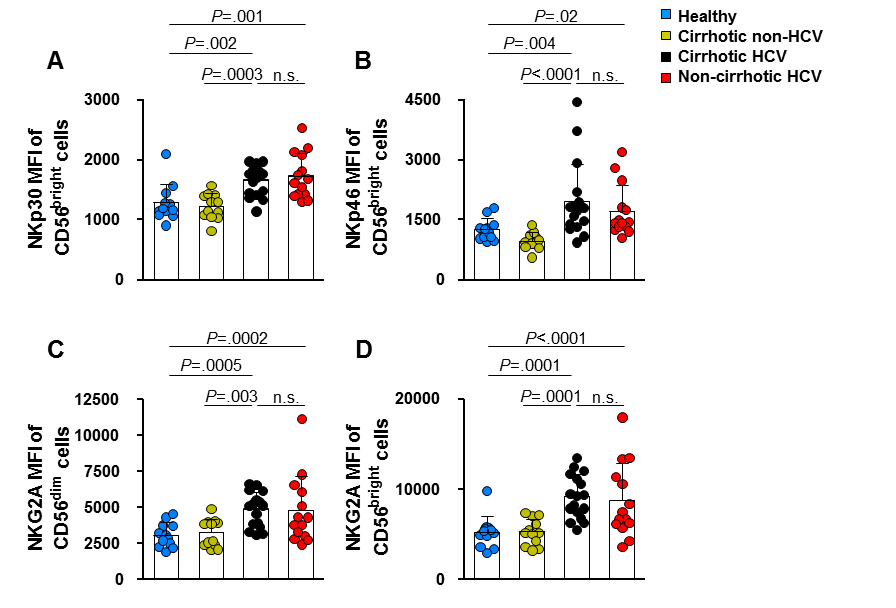


**Supplementary Figure 3.** **Baseline expression levels of NKp30, NKp46 and NKG2A receptors within NK subpopulations.** Expression levels, represented as mean fluorescent intensity (MFI), of NKp30 (A) and NKp46 (B) within the CD56^bright^ subset, and NKG2A within the CD56^dim^ (C) and CD56^bright^ cells (D) of 12 healthy individuals, 12 non-HCV cirrhotic controls, 17 HCV cirrhotic and 14 non-cirrhotic patients. Error bars indicate mean +SD; p-values were determined by Mann-Whitney U-test; n.s., not significant.

## Sl. Fig. 4


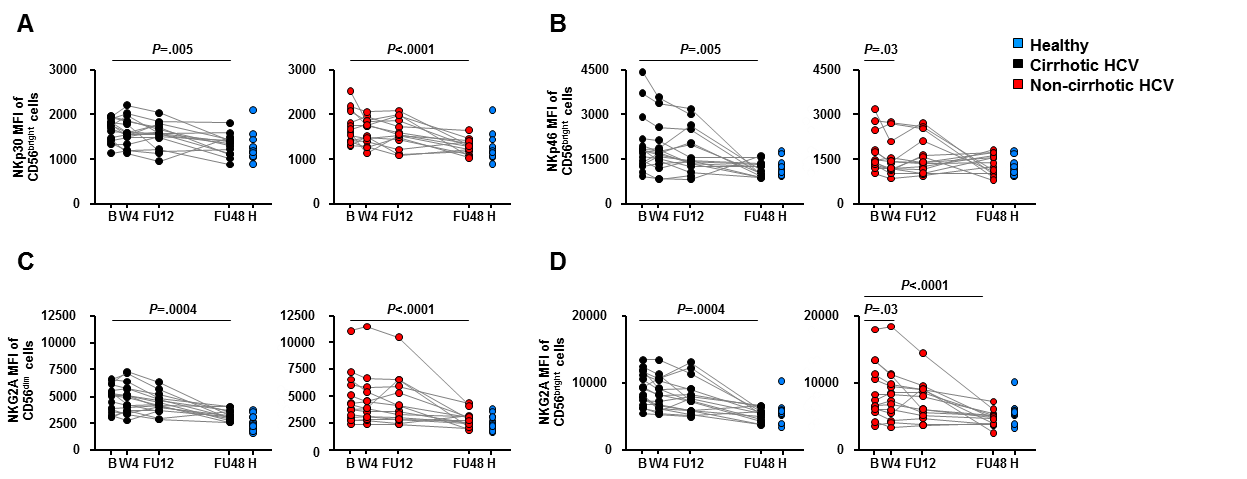


**Supplementary Figure 4. Longitudinal analysis of the** **expression levels of NKp30, NKp46 and NKG2A receptors within NK subpopulations.** Expression levels, represented as mean fluorescent intensity (MFI), of NKp30 (A) and NKp46 (B) within the CD56^bright^ subset, and NKG2A within the CD56^dim^ (C) and CD56^bright^ cells (D) of 17 HCV cirrhotic and 14 non-cirrhotic patients during and after IFN-free therapies. Error bars indicate mean +SD; p-values were determined by using Mixed Models for Repeated Measurements.

## Sl. Fig. 5


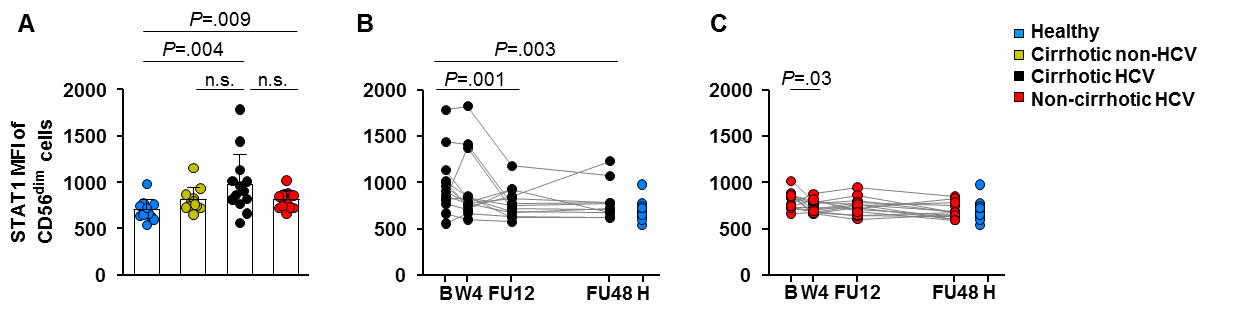


**Supplementary Figure 5. Normalization of the expression level of STAT1 in CD56^dim^ cells in HCV cirrhotic and non-cirrhotic patients.** (A) Baseline STAT1 MFI (mean fluorescent intensity) of CD56^dim^ cells in 12 healthy individuals, 12 non-HCV cirrhotic patients, 13 HCV cirrhotic and 14 non-cirrhotic patients. Error bars indicate mean ±SD; p-values were calculated by Mann-Whitney U test. Longitudinal analysis of the expression levels of STAT1 in CD56^dim^ cells during and after IFN-free therapies in (B) HCV cirrhotic and (C) non-cirrhotic patients. P-values were determined by using Mixed Models for Repeated Measurements.

## Sl. Fig. 6


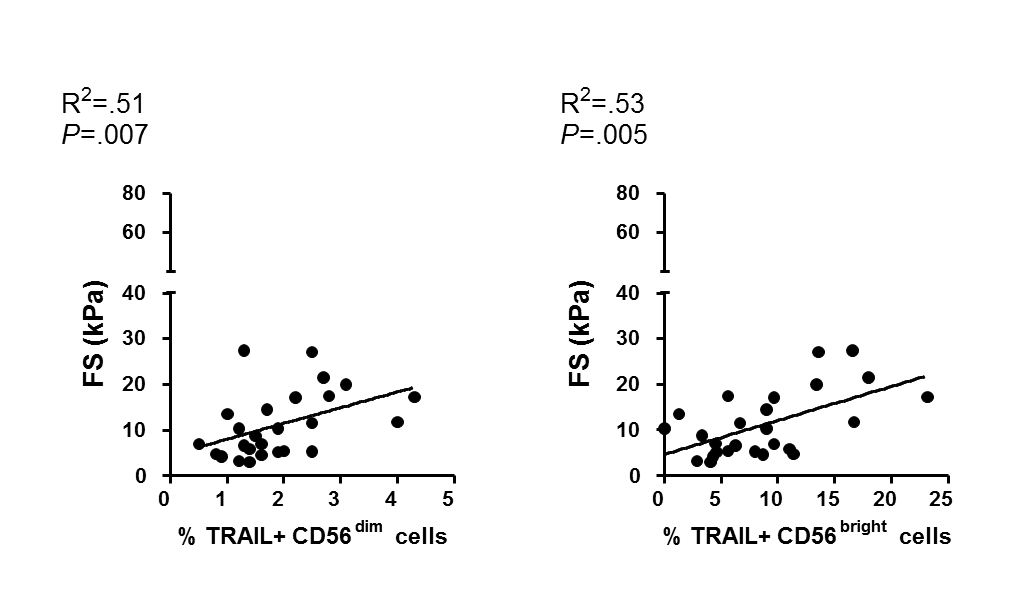


**Supplementary Figure 6. Liver stiffness values (FibroScan, FS) correlate with the frequencies of TRAIL at baseline in HCV patients.** Correlation between liver stiffness values, with the frequencies of TRAIL within CD56^dim^ (left) and CD56^bright^ (right) cells. P-values were determined by Spearman’s rank correlation coefficient.

**Sl. Fig. 7**

**
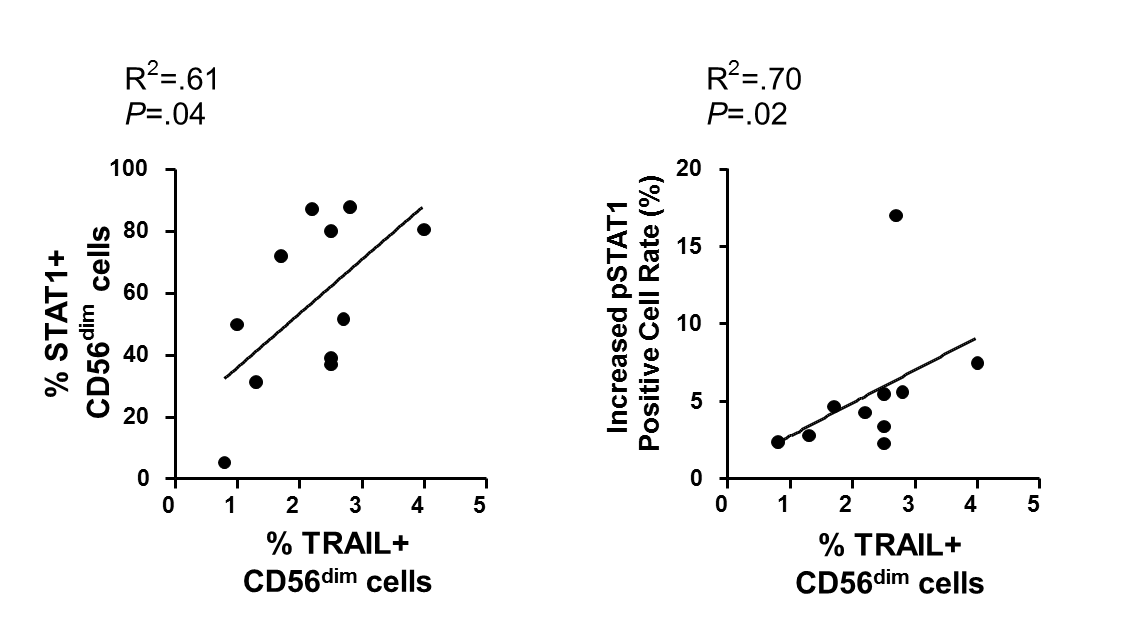
**

**Supplementary Figure 7. The link between STAT1/pSTAT1 and TRAIL expression is underscored in HCV-cirrhotic patients at baseline.** Correlation of the frequencies of TRAIL and STAT1 (left) or pSTAT1 (right) within CD56^dim^ cells. P-values were determined by Spearman’s rank correlation coefficient.

## Sl. Fig 8

**
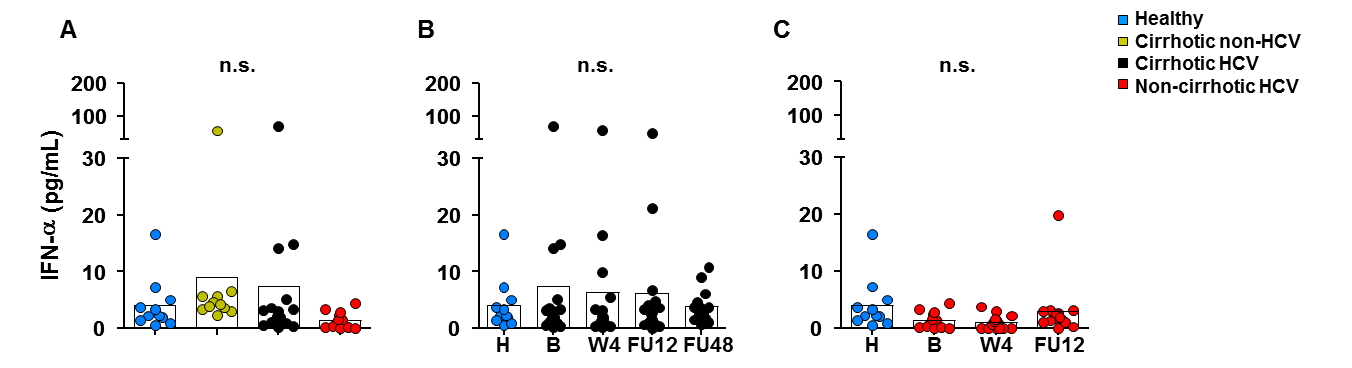
**

**Supplementary Figure 8. Analysis of serum IFN-α level.** (A) Baseline IFN-α level in 12 healthy individuals, 12 non-HCV cirrhotic controls, 17 HCV cirrhotic and 14 non-cirrhotic patients. Longitudinal analysis of IFN-α level in (B) HCV cirrhotic and (C) non-cirrhotic patients. Data are shown in bar columns indicate mean.

##
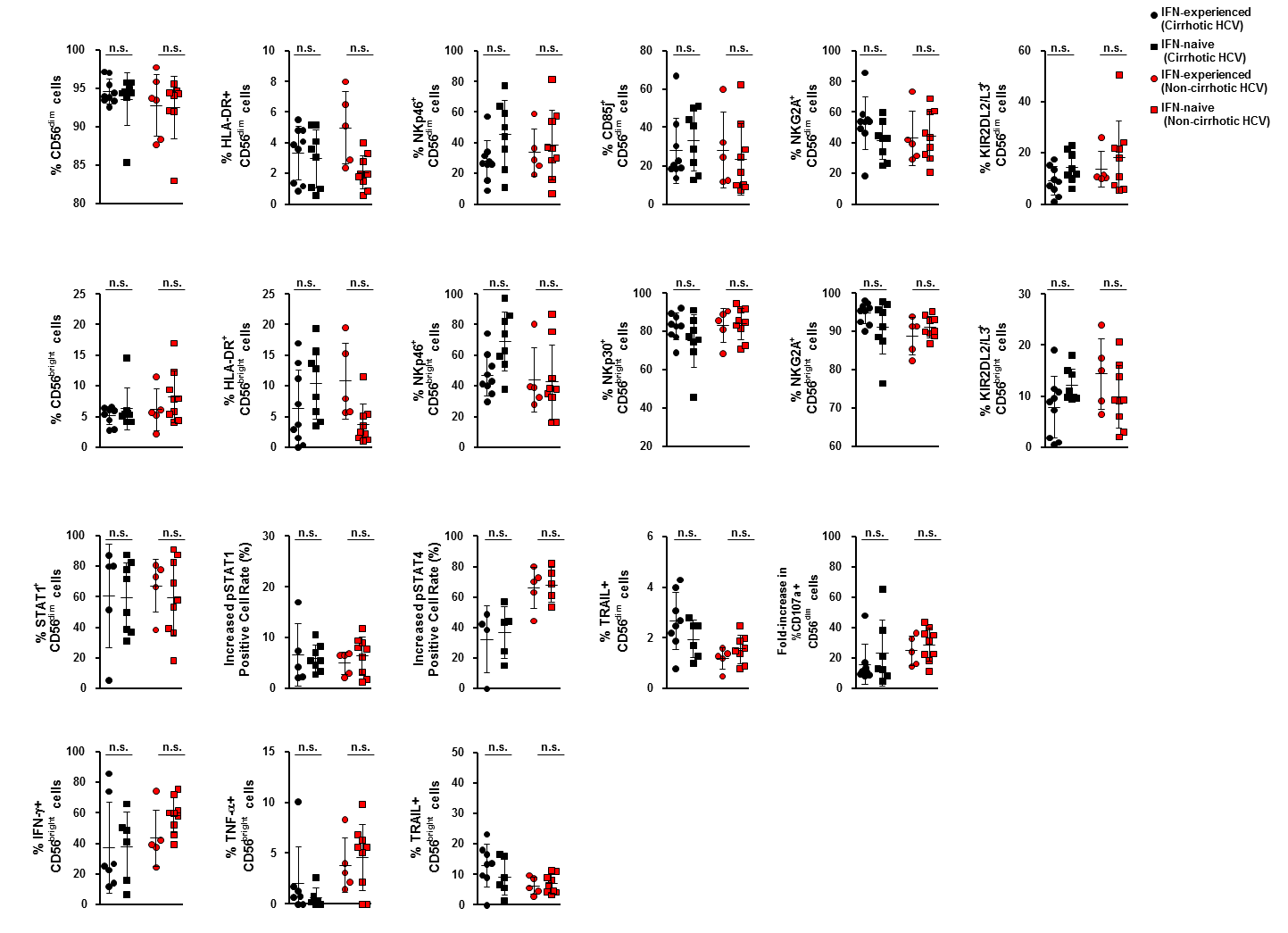
 Sl. Fig. 9

**Supplementary Figure 9. Baseline frequencies of phenotypical and functional markers in NK cells of HCV patients according to previous therapies.** Cirrhotic (black) and non-cirrhotic (red) patients are classified into naïve (square) or previous IFN-experienced (circle). *P*-values were calculated by Mann-Whitney U test. n.s., not significant.

##
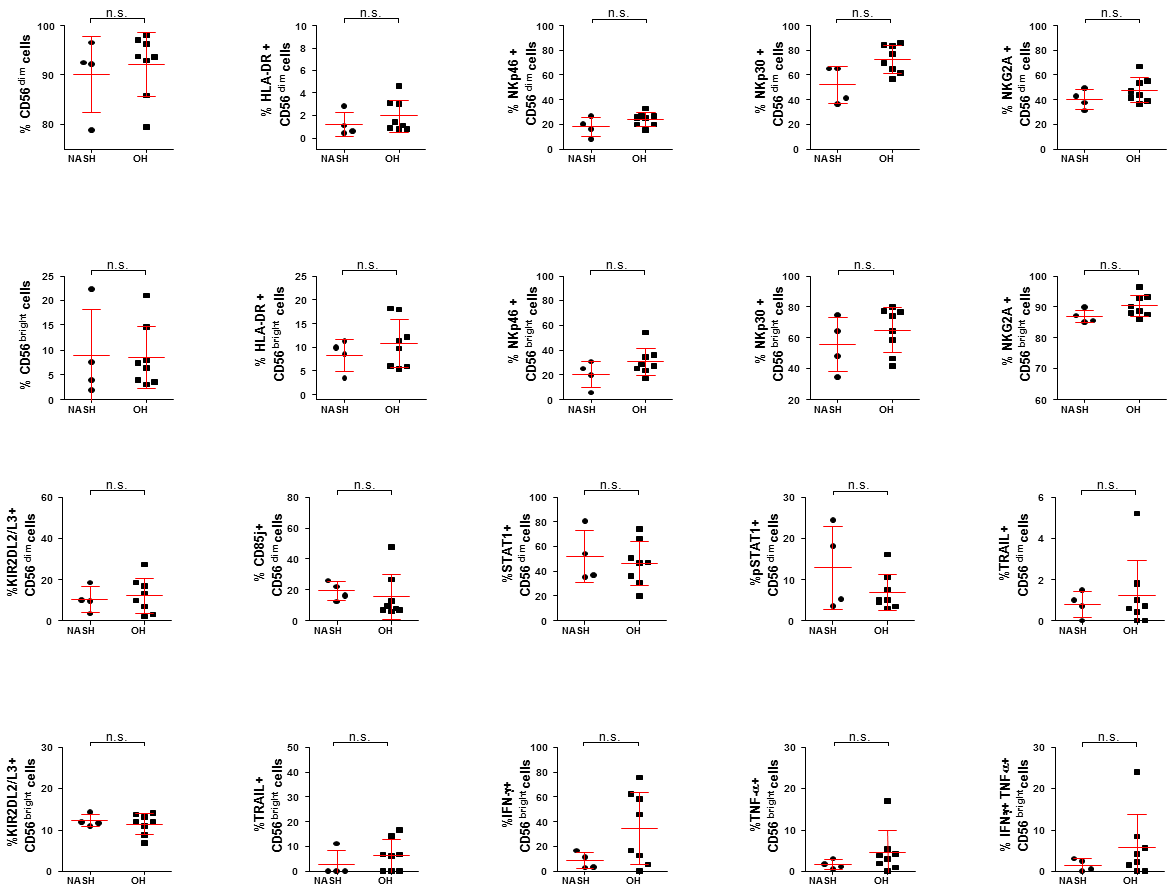
Sl. Fig. 10

**Supplementary Figure 10. Baseline frequencies of phenotypical and functional markers in NK cells of cirrhotic controls according to their etiology.** Cirrhotic controls are classified into alcoholic (square) or nonalcoholic steatohepatitis (NASH, circle) related cirrhosis. *P*-values were calculated by Mann-Whitney U test. n.s., not significant.

# Supplementary Tables

## Sl. Table 1

| Antigen | Fluorochrome | Clone | Company | Catalog number | Dilution |
| --- | --- | --- | --- | --- | --- |
| CD3, CD14, CD19 and CD20  (Lineage cocktail 3) | FITC | CD3 SK7, CD19 SJ25C1, CD20 L27, CD14 MφP9 | BD  Biosciences | 643510 | 1X |
| CD56 | PE | HCD56 | Biolegend | 318306 | 1X |
| NKp30 | AF647 | P30-15 | Biolegend | 325212 | 0.5X |
| NKp46 | PeCy7 | 9E2 | Biolegend | 331916 | 0.25X |
| KIR2DL2/L3 | PerCPCy5.5 | DX27 | Biolegend | 312614 | 0.5X |
| CD56 | PerCPCy5.5 | HCD56 | Biolegend | 318322 | 0.25X |
| HLA-DR | APC/Cy7 | L243 | Biolegend | 307618 | 0.25X |
| CD85j | AF647 | GHI/75 | Biolegend | 333709 | 1.5X |
| NKG2A | PE | REA110 | Miltenyi Biotec | 130-098-813 | 1X |
| CD107a | Pacific Blue | H4A3 | Biolegend | 328623 | 0.5X |
| STAT1 | PE | 1/Stat1 | BD Biosciences | 558537 | 0.03125X |
| pSTAT1 (pY701) | PerCPCy5.5 | 4a | BD Biosciences | 560113 | 0.25X |
| pSTAT4 (pY693) | PE | 38/p-Stat4 | BD Biosciences | 558249 | 1X |
| IFN-γ | PerCPCy5.5 | 4S.B3 | Biolegend | 502525 | 0.125X |
| TNF-α | AF647 | MAb11 | Biolegend | 502916 | 0.25X |
| TRAIL | APC | RIK-2 | Biolegend | 308210 | 0.5X |
| CD56 | V450 | MOPC-21 | BD Biosciences | 560361 | 1X |

**Supplementary Table 1**. List of antibodies used in multicolor flow cytometry analysis.
